# The effectiveness of interventions to prevent loneliness in the community-dwelling elderly population

**DOI:** 10.1093/eurpub/ckac131.489

**Published:** 2022-10-25

**Authors:** L Grillich, V Titscher, P Klingenstein, E Kostial, R Emprechtinger, I Sommer

**Affiliations:** 1 University for Continuing Education Krems, Department for Evidence-based Medicine and Evaluation, Krems, Austria; 2 University of Vienna, Department of Clinical and Health Psychology, Faculty of Psychology, Vienna, Austria; 3 Gesundheitsvorsorge GmbH, St. Pölten, Austria; 4 University for Continuing Education Krems, Faculty for Health and Medicine, Krems, Austria

## Abstract

**Background:**

Loneliness and social isolation have comparable health effects to widely acknowledged and established risk factors. Although the elderly are particularly affected, the effectiveness of interventions to prevent and/or mitigate social isolation and loneliness in the community-dwelling elderly is unclear. The aim of this review of reviews was to pool the findings of systematic reviews addressing the question of effectiveness.

**Methods:**

Ovid MEDLINE®, Health Evidence, Epistemonikos and Global Health (EBSCO) were searched from January 2017 to November 2021. Two reviewers independently assessed each systematic review in two consecutive steps based on previously defined eligibility criteria and appraised the methodological quality using AMSTAR 2. One author extracted data from both systematic reviews and eligible studies; another checked this. We conducted meta-analyses to pool the study results. We report the results of the random-effects and common-effect models.

**Results:**

We identified 5 systematic reviews containing a total of 30 eligible studies, 16 with a low or moderate risk of bias. Our random-effects meta-analysis indicates an overall SMD effect of 0.63 [95% CI: -0.10; 1.36] for loneliness and was unable to detect an overall effect of the interventions on social support [SMD: 0.00; 95% CI: -0.11; 0.12].

**Discussion:**

The results show interventions can potentially reduce loneliness in the non-institutionalised, community-dwelling elderly population living at home. As confidence in the evidence is low, rigorous evaluation is recommended.

**Key messages:**

• The meta-analysis indicates that psychosocial interventions have the potential to reduce loneliness in the community-dwelling elderly population.

• As confidence in the evidence is low, healthcare providers should only implement interventions that are, firstly, based on a sound theory of change and, secondly, subject to rigorous evaluation.

